# Benefits of stereotactic ablative radiotherapy combined with incomplete transcatheter arterial chemoembolization in hepatocellular carcinoma

**DOI:** 10.1186/s13014-016-0597-7

**Published:** 2016-02-19

**Authors:** Eun Kyung Paik, Mi-Sook Kim, Won Il Jang, Young Seok Seo, Chul-Koo Cho, Hyung Jun Yoo, Chul Ju Han, Su Cheol Park, Sang Bum Kim, Young Han Kim

**Affiliations:** Department of Radiation Oncology, Korea Cancer Center Hospital, Korea Institute of Radiological and Medical Sciences, 75 Nowon-ro, Nowon-gu, Seoul, Republic of Korea; Department of Internal Medicine, Korea Cancer Center Hospital, Korea Institute of Radiological and Medical Sciences, Seoul, Republic of Korea; Department of Surgery, Korea Cancer Center Hospital, Korea Institute of Radiological and Medical Sciences, Seoul, Republic of Korea; Department of Radiology, Korea Cancer Center Hospital, Korea Institute of Radiological and Medical Sciences, Seoul, Republic of Korea

**Keywords:** Hepatocellular carcinoma, Radiotherapy, Stereotactic ablative radiotherapy, Transcatheter arterial chemoembolization

## Abstract

**Background:**

This study aimed to evaluate the effect of stereotactic ablative radiotherapy (SABR) after incomplete transcatheter arterial chemoembolization (TACE) in hepatocellular carcinoma (HCC) patients.

**Methods:**

The study enrolled 178 HCC patients initially treated with TACE between 2006 and 2011. Patients were included if they had Barcelona Clinic Liver Cancer stage 0 or A, ≤3 nodules with a total sum of longest diameter ≤10 cm, Child-Turcotte-Pugh score of ≤7, no major vessel invasion, and no extra-hepatic metastases.

**Results:**

Twenty-four patients achieved a complete response to TACE (group 1). Among those with incomplete response, 47 patients received other curative treatments (group 2), 37 received SABR (group 3), and 70 received non-curative treatments (group 4). The 2–year overall survival (OS) rates for groups 1, 2, 3, and 4 were 88 %, 81 %, 73 %, and 54 %, respectively. The corresponding 5-year OS rates were 50 %, 58 %, 53 %, and 28 %, respectively.

**Conclusions:**

Patients treated with SABR after incomplete TACE had similar survival outcomes to those achieving complete response to TACE or receiving curative treatments. However, patients receiving non-curative treatments had significantly lower survival rates than the other groups. Therefore, if SABR was indicated at the initial diagnosis, it might be recommended after TACE failure.

## Background

The primary treatment for hepatocellular carcinoma (HCC) is surgery, including hepatic resection and liver transplantation, which results in 5-year survival rates of 30–70 % [1]. However, <20 % of HCC patients are eligible for surgery. In unresectable cases, local therapies such as radiofrequency ablation (RFA) or percutaneous ethanol injection (PEI) are reportedly effective, but not all patients are eligible for these treatments due to the tumor’s percutaneous inaccessibility, its invisibility via ultrasonography, or bleeding risks. Transcatheter arterial chemoembolization (TACE) has been widely used as the first-line non-curative therapy for HCC cases that are non-surgical or unsuitable for local ablative therapies [2]. However, TACE alone rarely produces a complete response, and additional treatments are often required. Multiple repetitive sessions of TACE have been widely performed in Korea, but such treatments deteriorate liver function, increase TACE-related adverse effects, and offer less therapeutic efficacy due to vascularity decrease [3]. Therefore, various modalities such as RFA, PEI, sorafenib, conventional radiotherapy (RT), and stereotactic ablative radiotherapy (SABR) have been suggested in addition to TACE, but a definitive guideline has not been established [4, 5].

The role of RT in HCC is limited owing to the liver’s low tolerance to radiation and the risk of radiation-induced liver damage [6, 7]. However, recent radiotherapeutic developments have gradually expanded the indications for external beam radiotherapy from palliative to curative with high doses of radiation safely delivered to the tumor while avoiding adverse effects to the liver function. In several studies on three-dimensional conformal radiotherapy (3D-CRT) to primary HCC, substantial effects of RT have been observed [8–10]. With the introduction of SABR, it is now possible to accurately deliver more radiation doses to tumors using fewer fractions while sparing the normal liver tissue [11, 12]. Recent clinical data have demonstrated the feasibility of SABR for HCC treatment with high local control (LC) and overall survival (OS) rates and low treatment-related severe toxicities [13–21].

However, there remains a lack of randomized studies comparing the effects of TACE combined with RT and those of TACE alone. Although several phase II trials of SABR have reported outstanding results, including LC rates of > 90 % and 5-year OS of > 50 % in well selected cases of HCC, RT is still not endorsed as a curative treatment option for HCC in most guidelines or consensus strategies [1, 22, 23]. Therefore, this retrospective study aimed to provide the basis for initiating a randomized trial to investigate whether a combination of SABR and TACE would improve the long-term OS compared with TACE alone. The survival outcomes of TACE plus SABR were compared with those of other treatment modalities combined with TACE or TACE alone.

## Methods

### Patients

Between January 2006 and December 2011, a total of 832 consecutive HCC patients were treated with TACE at Korea Institute of Radiological and Medical Sciences. Of those, patients receiving curative treatments such as surgery or RFA at initial diagnosis were excluded, and only those initially treated with TACE were included in this study. All patients’ medical records were retrospectively reviewed. A total of 178 patients met the following eligibility criteria: (1) Barcelona Clinic Liver Cancer (BCLC) stage 0 or A, (2) single or multiple lesions (≤3 nodules), (3) each tumor measuring ≤10 cm at the longest diameter (LD), with the sum of LD being ≤10 cm, (4) Child-Turcotte-Pugh (CTP) score of ≤7, (5) no major vessel invasion, and (6) no extrahepatic metastases. The exclusion criteria included (1) diffuse infiltrative tumor type, (2) liver cirrhosis-associated complications such as uncontrolled ascites or encephalopathy, (3) severe co-morbidities, (4) previous RT to the upper abdomen, and (5) other malignancies within 5 years. These are our institutional criteria for curative SABR in HCC patients [4, 19]. This study was approved by our institutional review board.

### TACE

TACE was performed with an infusion of a lipiodol and doxorubicin mixture. Tumor response to TACE was evaluated using computed tomography (CT) 1 month after TACE. Although the definition of TACE failure has been proposed by the Japan Society of Hepatology [23], there is no internationally accepted consensus on the definition of TACE failure or TACE refractoriness criteria. In our study, incomplete response to TACE was defined as disease progression, incomplete tumor filling by the lipiodol-doxorubicin mixture on CT images. Disease progression included residual viable tumors, progression of existing lesions, or development of new lesions. Patients’ final response to TACE was assessed by a hepatologist. Although current evidence suggests that 1 cycle of TACE may not be sufficient for effective treatment and repeating TACE prolongs survival [24], no guidelines are available on the criteria for repeating TACE. In this study, if TACE yielded a complete response, no additional treatment was given, and the patient was followed up by regular evaluations. In cases of incomplete response, additional TACE was performed, or a change in treatment strategy was conducted.

### SABR

SABR was performed as previously described [4, 19]. Patients were treated using either a CyberKnife (Accuray Inc., Sunnyvale, CA, USA) or a RapidArc (Varian Medical Systems, Palo Alto, CA, USA) system. Gold fiducials inserted near the spine closest to the lesion were used for tracking in patients treated with CyberKnife. For patients treated with RapidArc, gold fiducials inserted in the liver near the tumor or lipiodol uptake were used as markers for image guidance with the cone-beam CT. To compensate for breathing motion, simulation CT with abdominal compression was performed, and both slow and helical CT images were obtained with contrast medium. The gross tumor volume (GTV) was defined in the slow CT set as the viable tumor with the contrast medium uptake including the embolization material. These slow CT images included the respiratory movement of the target, therefore the GTV used for planning was larger than the actual gross tumor and was referred to as the internal target volume (ITV). To better delineate the tumor volumes, magnetic resonance imaging (MRI) images were used as references on a regular basis. A margin of 0–4 mm was added to the ITV for the planning target volume (PTV). SABR doses were prescribed at an isodose line (70–80 % of the maximum dose) that covered at least 97 % of the PTV.

Treatment doses were determined according to previously described protocols [4, 19, 20]. A total dose of 40–60 Gy (median 56 Gy) in 3–5 fractions was prescribed. Briefly summarizing the previous protocols, the final prescribed doses were 60 Gy in 3 fractions, but the dosages were reduced by 0.5 or 1 Gy per fraction until the normal tissue constraints were allowed. For the normal liver, at least 700 ml of normal liver (entire liver minus the cumulative GTV) should not receive a total dose ≥ 17 Gy in 3 fractions. For the spinal cord, the maximum dose should not exceed 18 Gy in 3 fractions. For the esophagus, the maximum dose should not exceed 24 Gy in 3 fractions. In addition, although other normal tissue constraints were not considered, dosages to the kidney, intestine, and stomach were restricted to the lowest levels possible.

### Evaluation and statistical analysis

Baseline patient and tumor characteristics were compared among groups using the one-way analysis of variance or the Kruskal-Wallis test as appropriate. Survival was calculated from the date of the initial TACE using the Kaplan-Meier method, and intergroup comparisons were performed using the log-rank test. Univariate analysis was performed using the log-rank test to identify the significant prognostic factors for survival. The Cox regression model was applied to all potentially significant variables for the multivariate analysis. For all analyses, two-sided tests of significance were used with *P* values <0.05 considered significant. All statistical analyses were performed using the Statistical Package for the Social Sciences (version 14.0; SPSS, Inc. Chicago, IL, USA).

## Results

### Baseline patient characteristics

**Fig. 1:**
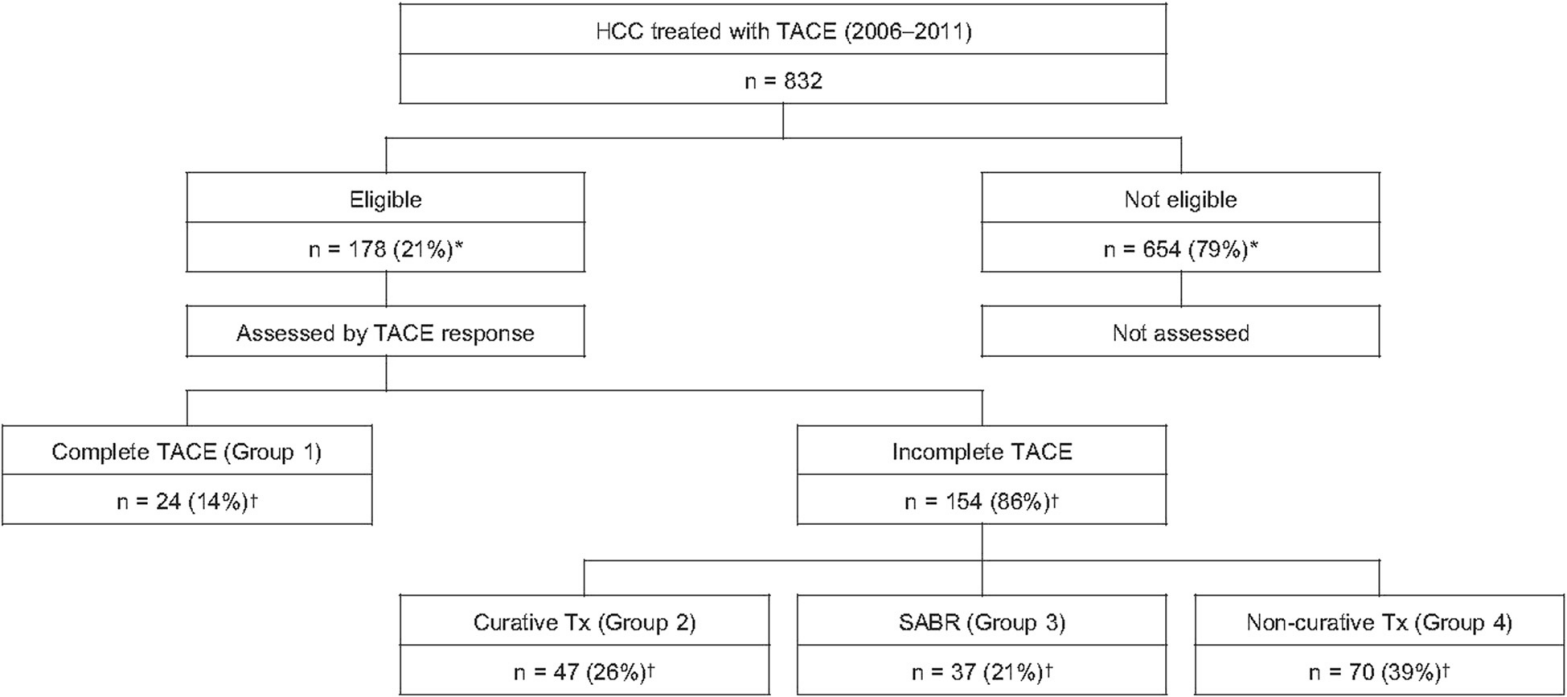
Study design flowchart. Twenty-one percent of the patients treated with TACE met the eligibility criteria, which is also the criteria for SABR with curative intent in our institute. These patients were divided into 4 groups according to the further treatment received. Abbreviations: HCC, hepatocellular carcinoma; TACE, transcatheter arterial chemoembolization; Tx, treatment; SABR, stereotactic ablative radiotherapy. * Percentage of patients from the total number of patients receiving TACE (*n* = 832). † Percentage of patients from those meeting the eligibility criteria (*n* = 178)

Of the 832 consecutive HCC patients treated with TACE, 178 met all eligibility criteria. Our enrollment suggests that among patients currently treated with TACE, approximately 21 % (or higher if those initially treated with surgery or other local ablative therapies are included) may be eligible for SABR. Twenty-four (14 %) patients achieved a complete response to TACE as the primary treatment (group 1; complete TACE group). The remaining 154 (86 %) patients showed incomplete response and were further treated with other modalities. Of these, 47 (26 %) patients further received curative treatments (group 2; incomplete TACE + curative Tx group) such as surgery, RFA, or PEI; 37 (21 %) were treated with SABR (group 3; incomplete TACE + SABR group); and 70 (39 %) received non-curative treatments (group 4; incomplete TACE + non-curative Tx group) such as repeated TACE, sorafenib, and chemotherapy (Fig. 1). Among the 47 group 2 patients, 35 (75 %) received surgery including resection and liver transplantation, 11 (23 %) received RFA, and 1 (2 %) received PEI. Among the 70 group 4 patients, 66 (95 %) received repeated TACE, 3 (4 %) received sorafenib, and 1 (1 %) received chemotherapy. The median number (range) of previous TACE sessions performed for groups 1, 2, 3, and 4 patients were 1 (1–4), 1 (1–5), 2 (1–6), and 2 (1–13), respectively.

**Fig. 2:**
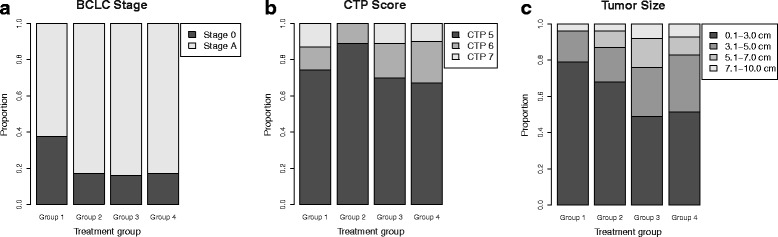
Tumor characteristics of each treatment group. **a** By Barcelona Clinic Liver Cancer (BCLC) stage; (**b**) By Child-Turcotte-Pugh (CTP) score; (**c**) By tumor size

No significant differences in age, sex, BCLC stage, and tumor size were observed among the 4 groups (Table 1). However, statistically significant differences in CTP scores were observed between groups 2 and 4 (*P* = 0.004). Furthermore, patient characteristics did not differ among groups 1, 2, and 3, or between groups 3 and 4. Nonetheless, group 1 patients had relatively lower BCLC stages and smaller tumors than the other groups, whereas those in group 2 had lower CTP scores and smaller tumors (Fig. 2).

**Table 1:** Number of patients (%) for patient and tumor characteristics of each treatment group

Characteristics	Complete TACE	Incomplete TACE			*P*-value
		Curative Tx	SABR	Non-curative Tx	
Age					0.622
Range (median, years)	41–83 (55)	35–77 (60)	42–74 (59)	24–84 (59)	
Sex					0.426
Male	14 (58)	33 (70)	29 (78)	49 (70)	
Female	10 (42)	14 (30)	8 (22)	21 (30)	
BCLC stage					0.135
0	9 (38)	8 (17)	6 (16)	12 (17)	
A	15 (63)	39 (83)	31 (84)	58 (83)	
CTP score					0.040
5	18 (75)	42 (89)	26 (70)	47 (67)	
6	3 (13)	5 (11)	7 (19)	16 (23)	
7	3 (13)	0 ( 0)	4 (11)	7 (10)	
Tumor size					0.073
Range (median, cm)	0.5–8 (2.1)	1–8 (2.8)	0.8–10 (3.4)	0.5–10 (3.8)	
0.1–3.0 cm	19 (79)	32 (68)	18 (49)	36 (51)	
3.1–10.0 cm	5 (21)	15 (32)	19 (51)	34 (49)	

*Abbreviations*: *TACE* transcatheter arterial chemoembolization, *Tx* treatment, *SABR* stereotactic ablative radiotherapy, *BCLC* Barcelona Clinic Liver Cancer, *CTP* Child-Turcotte-Pugh

### Overall survival

**Fig. 3:**
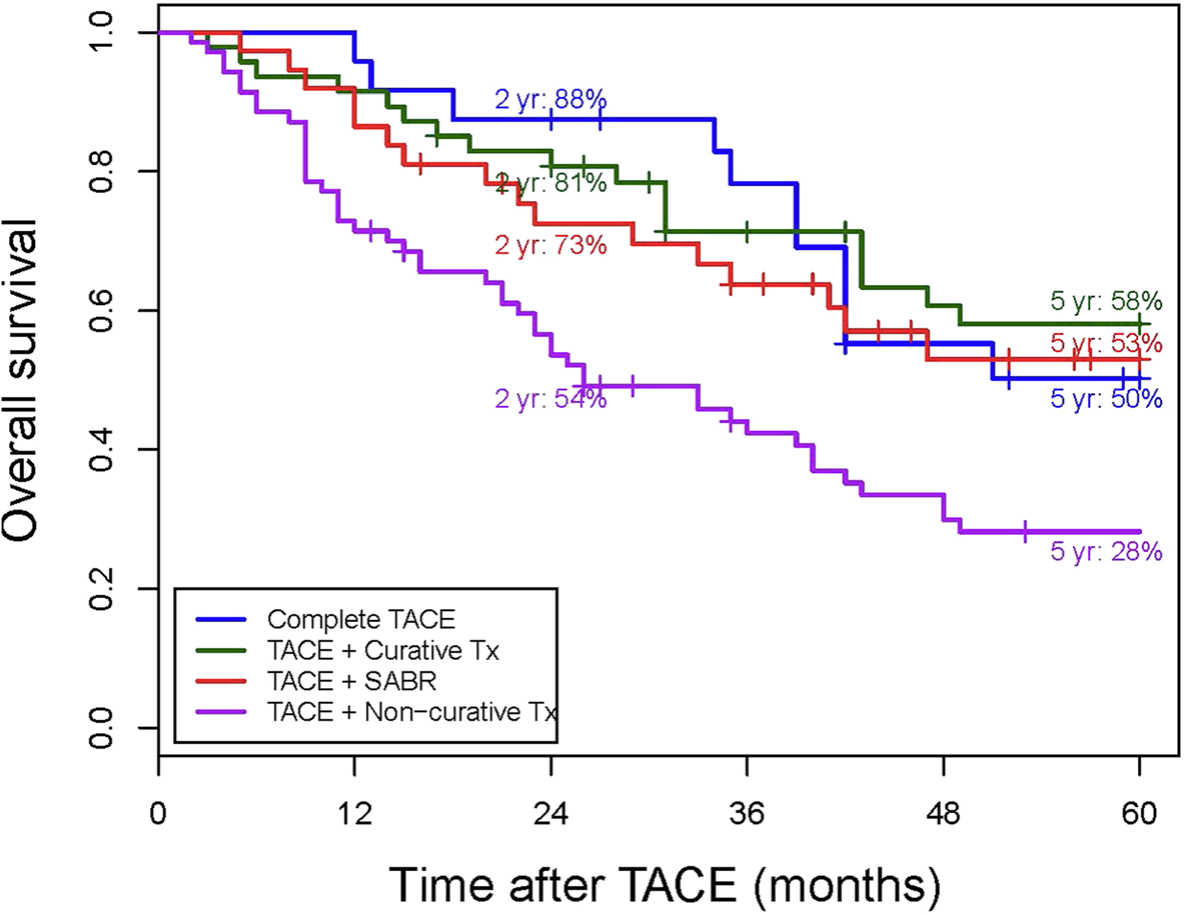
Patients’ overall survival for each treatment group from the time of the first TACE treatment. No significant differences in overall survival rates were observed among groups 1, 2, and 3. However, group 4 showed a significantly inferior overall survival rate compared with the other 3 groups. Abbreviations: TACE, transcatheter arterial chemoembolization; Tx, treatment; SABR, stereotactic ablative radiotherapy

The median follow up duration after the initial TACE for all patients was 35 months (range, 2–83 months). The OS rates at 2 and 5 years were 88 % and 50 % for group 1, 81 % and 58 % for group 2, 73 % and 53 % for group 3, and 54 % and 28 % for group 4, respectively (Fig. 3). No significant differences in OS rates were observed among groups 1, 2, and 3. However, group 4 showed a significantly different OS rate compared with the other 3 groups (vs. group 1, *P* = 0.010; vs. group 2, *P* = 0.001; vs. group 3, *P* = 0.040; Table 2).

**Table 2:** Prognostic factors for overall survival

Factor	5-year OS (%)	Univariate analysis	Multivariate analysis
		*P*-value	HR (95 % CI)
Treatment option			
Complete TACE	50		
TACE + Curative Tx	58	0.843	1.100 (0.521–2.324)
TACE + SABR	53	0.493	1.207 (0.573–2.545)
TACE + Non-curative Tx	28	0.010	2.421 (1.248–4.498)
BCLC stage			
0	54		
A	42	0.139	-
CTP score			
5	48		
6	33	0.128	1.037 (0.622–1.729)
7	29	0.013	2.229 (1.147–4.332)
Tumor size (cm)			
0.1–3.0	49		
3.1–10.0	32	0.008	-

*Abbreviations*: *OS* overall survival, *TACE* transcatheter arterial chemoembolization, *Tx* treatment, *SABR* stereotactic ablative radiotherapy, *BCLC* Barcelona Clinic Liver Cancer, *CTP* Child-Turcotte-Pugh

In univariate analysis, CTP score, tumor size, and treatment options were identified as significant prognostic factors for OS (Table 2). A CTP score of 7 was associated with significantly lower survival rates than CTP scores of 5 or 6. Tumors >3 cm had significantly lower survival rates than those <3 cm. In multivariate analysis, CTP score and treatment options were identified as significant prognostic factors. Group 4 showed significantly lower survival rates than the other 3 groups, but no significant differences were observed among groups 1, 2, and 3.

## Discussion

In previous phase I and phase II studies conducted at our institute [4, 19], the feasibility of SABR for the treatment of primary HCC with incomplete response to TACE has been demonstrated with a 2-year LC rate of 94.6 % and a 2-year OS rate of 68.7 %. In the long term follow up study [20], LC and OS rates at 5 years were 82 % and 39 %, respectively. Furthermore, at the last follow-up (4.5 years), patients receiving high-dose SABR (>54 Gy) reported a 100 % LC rate and a 68 % OS rate, which were comparable to RFA outcomes. Based on the results of these studies of SABR, HCC cases with a CTP score of 5–7, total sum of tumor size < 10 cm, and ≤ 3 nodules seem to be feasible for curative SABR. Therefore, to investigate whether the addition of SABR to TACE offers a survival benefit to HCC patients with incomplete response to TACE, we enrolled those meeting the above-mentioned eligibility criteria. In this study, the addition of SABR after incomplete TACE demonstrated significant survival benefits compared with additional non-curative treatments such as repeated TACE, sorafenib, and chemotherapy. Moreover, it also showed similar survival outcomes to those of the good prognostic groups, such as patients with complete response to TACE or those undergoing surgery with incomplete TACE.

TACE is widely adopted as the current standard of care for HCC patients at BCLC intermediate stage. Additionally, it might be indicated for patients with early stage HCCs that are unresectable or ineligible for local ablative therapies such as RFA or PEI due to tumor location or other medical conditions. Although complete response to TACE has not yet been defined in radiological findings, compact lipiodolisation is an important goal of TACE. Cabibbo et al. [25] reported that a complete radiological response after TACE significantly increases OS, and thus proposed utilizing the observation as a surrogate treatment endpoint. Kim et al. [26] indicated that compact lipiodol uptake following TACE predicted favorable survival outcomes for unresectable HCC. The 1-year and 5-year OS rates of patients with compact lipiodolisation were 93 % and 52 %, respectively, versus 61 % and 17 %, respectively, in patients with noncompact lipiodolisation. Similarly, in our current study, the OS rates of group 1 patients with complete response to TACE at 1 and 5 years were 96 % and 53 %, respectively. In addition, only 14 % of the enrolled patients in this study achieved a complete response after 1–4 sessions of repeated TACE. Furthermore, approximately 40 % of the patients were in stage 0, and almost 80 % had tumors <3 cm, suggesting that group 1 patients had relatively lower BCLC stages and smaller tumors than those in the incomplete response groups. Therefore, although group 3 patients, who received SABR after incomplete response to TACE, might have had poorer prognostic factors than group 1 patients, similar OS rates were observed between the 2 groups.

Since a compact radiological response after TACE significantly increases OS, technical improvements and refinements have been described for transarterial administrative methods [3, 27]. However, many patients still experience incomplete response after TACE, especially in cases of tumors >3 cm [28]. Several studies [29–32] have shown that the combination of TACE and other modalities, mainly surgery, RFA, PEI, or RT, was associated with higher survival rates. According to a prospective cohort study by Lee et al. [30], in which surgical resection after primary TACE was compared with TACE alone, the OS rate was significantly higher for the surgical resection group than for the TACE alone group (5-year OS rate, 56 % vs. 23 %). Studies on TACE plus RFA showed that patients in the TACE plus RFA group had better LC rates than patients in the TACE alone group [31], and better OS rates than patients in the RFA alone group [32]. TACE plus RFA have also been reported to provide similar OS rates to those achieved with surgical resection [33]. Several randomized trials were conducted to compare TACE plus PEI and TACE alone [34–36], suggesting that TACE plus PEI performed better than either TACE or PEI alone, and the reported 3-year OS rates for TACE plus PEI were approximately 35 %–65 %. In our current study, group 2 patients underwent surgery, RFA, or PEI after incomplete TACE. This group only included patients with CTP class A. Our findings suggested that TACE might have been performed as a bridge therapy in patients who were initially eligible for curative treatments and subsequently underwent additional surgery. Also, group 2 patients had significantly lower CTP scores than the non-curative treatment group 4 patients. Therefore, group 2 patients might initially have had good prognostic factors, and our results also indicated a significant survival benefit in this group compared with group 4. Additionally, although not statistically significant, group 2 consisted of patients with relatively lower CTP scores compared to group 3, and smaller tumors compared to groups 3 and 4. Therefore, although group 3 patients might have had poorer prognostic factors than group 2 patients, similar OS rates were observed.

The benefit of additional 3D-CRT to incomplete TACE over TACE alone in unresectable HCC has also been reported. Meng et al. [10] conducted a meta-analysis of 17 trials, including 5 randomized controlled and 12 non-randomized controlled studies. Although TACE plus RT showed significantly improved survival and tumor response compared with TACE alone in this meta-analysis, none of the 5 randomized trials reported the random allocation sequence in detail, and most of the original studies included were published in Chinese, making general acceptance challenging. On the other hand, in a retrospective study, Shim et al. [5] reported that a combination of 3D-CRT and incomplete TACE significantly improved survival rates compared with TACE alone. Their study included single tumors ≥5 cm, and the 2-year OS rates of the TACE plus RT and TACE alone groups were 37 % and 14 %, respectively. The combination of SABR and TACE has previously been studied by Honda et al. [21]. Their results showed that SABR combined with TACE was safe and effective for loco-regional treatments, increasing both LC and OS compared with TACE alone. The 1-year and 3-year OS rates for the SABR group were 100 % and 100 %, respectively, whereas the corresponding rates for the TACE group were 89 %, and 66 %, respectively. Although excellent OS rates were reported, patient selection was very strict, with only solitary tumors and small tumors ≤3 cm included. Our results also indicated a significant survival benefit of TACE plus SABR (group 3) over TACE alone (group 4) with similar baseline characteristics between the 2 groups.

There is not enough evidence to establish any combination therapy as a standard treatment for HCC after incomplete TACE. In our study, combining SABR to TACE offered similar survival outcomes to TACE and curative treatments, and survival benefits over repeated TACE, suggesting that SABR might be recommended as a treatment modality after TACE failure. Furthermore, SABR, being a noninvasive procedure, might be more advantageous than other invasive curative modalities such as surgery, RFA, or PEI. However, since this was a retrospective study, patients were not controlled with respect to variable prognostic factors. Additionally, the dose and fractionation schedules of SABR and the number of previous TACE sessions prior to SABR are still not well defined and are yet to be determined. Therefore, further randomized trials are needed to validate our results before SABR can be recommended routinely. Hence, based on the results of this study, a multicenter randomized controlled trial is being planned to investigate the potential benefits of SABR as an alternative modality in the treatment of HCC after incomplete TACE.

## Conclusions

Patients treated with SABR after incomplete TACE had similar survival outcomes to those achieving complete response to TACE or those receiving curative treatments such as surgery, RFA, or PEI after incomplete TACE. The addition of SABR also had a significant survival benefit compared to repetitive TACE treatments. The present retrospective study showed that about 20 % of the patients initially treated with TACE met the eligibility criteria for curative SABR treatments, and may benefit from further treatments of SABR. Therefore, SABR might be considered an alternative treatment modality following TACE failure.

## Abbreviations

3D-CRT: three-dimensional conformal radiotherapy
AFP: alpha-fetoprotein
BCLC: Barcelona Clinic Liver Cancer
CT: computed tomography
CTP: Child-Turcotte-Pugh
GTV: gross tumor volume
HCC: hepatocellular carcinoma
ITV: internal target volume
LC: local control
LD: longest diameter
OS: overall survival
PEI: percutaneous ethanol injection
PTV: planning target volume
RFA: radiofrequency ablation
RT: radiotherapy
SABR: stereotactic ablative radiotherapy
TACE: transcatheter arterial chemoembolization
